# Supplementary material for: κ-Selenocarrageenan Oligosaccharides Prepared by Deep-Sea Enzyme Alleviate Inflammatory Responses and Modulate Gut Microbiota in Ulcerative Colitis Mice
Source: Int J Mol Sci. 2023 Feb 28;24(5):4672. doi: 10.3390/ijms24054672 (PMC10003262; doi:10.3390/ijms24054672)
Supplement: Supplementary file 1 [file ijms-24-04672-s001.zip › Figure caption.pdf]

Figure S1. Multiple sequence alignment of SeCar with characterized GH16 κ-carrageenases. The star represents amino acid conserved residues.

Figure S2. SDS-PAGE of purified SeCar. Lane M, protein marker; Lane 1, purified SeCar.

Figure S3. Chemical composition analysis and structure prediction of KSCOs. (A) HPGPC chromatogram; (B) ESI-MS analysis. DA, 3, 6-anhydrogro-α-1, 4 linked-D-galactose; G4, β-1, 3-D-galactose-4-sulfate; (C) the predicted structure of selenium-galactobioses.

Figure S4. TLC analysis of of κ-selenocarrageenan hydrolysis products. Lane M, standard samples of carabiose, caratetraose and carahexaose; Lane 1, substrate before hydrolysis; Lane 2, substrate after hydrolysis.

Figure S5. Alpha-diversity of gut microbiota and rarefaction curves. (A) Ace index; (B) Shannon index; (C) Simpson index; and (D) rarefaction curves based on the operational taxonomic unit (OTU) level; each line represents one sample, and lines of the same color represent samples from the same group.
