# Supplementary material for: κ-Selenocarrageenan Oligosaccharides Prepared by Deep-Sea Enzyme Alleviate Inflammatory Responses and Modulate Gut Microbiota in Ulcerative Colitis Mice
Source: Int J Mol Sci. 2023 Feb 28;24(5):4672. doi: 10.3390/ijms24054672 (PMC10003262; doi:10.3390/ijms24054672)
Supplement: Supplementary file 1 [file ijms-24-04672-s001.zip › Table S1.pdf]

Table S1 Molecular weight distribution of hydrolysis products

Peak time (min)	Molecular weight (Da)	Proportion (%)
18.795	8217.73	2.54
23.239	1379.49	37.14
23.929	1045.64	8.23
24.544	816.82	31.69
26.191	421.59	5.25
27.322	267.70	15.15
