# Supplementary figures and images for: κ-Selenocarrageenan Oligosaccharides Prepared by Deep-Sea Enzyme Alleviate Inflammatory Responses and Modulate Gut Microbiota in Ulcerative Colitis Mice
Source: Int J Mol Sci. 2023 Feb 28;24(5):4672. doi: 10.3390/ijms24054672 (PMC10003262; doi:10.3390/ijms24054672)

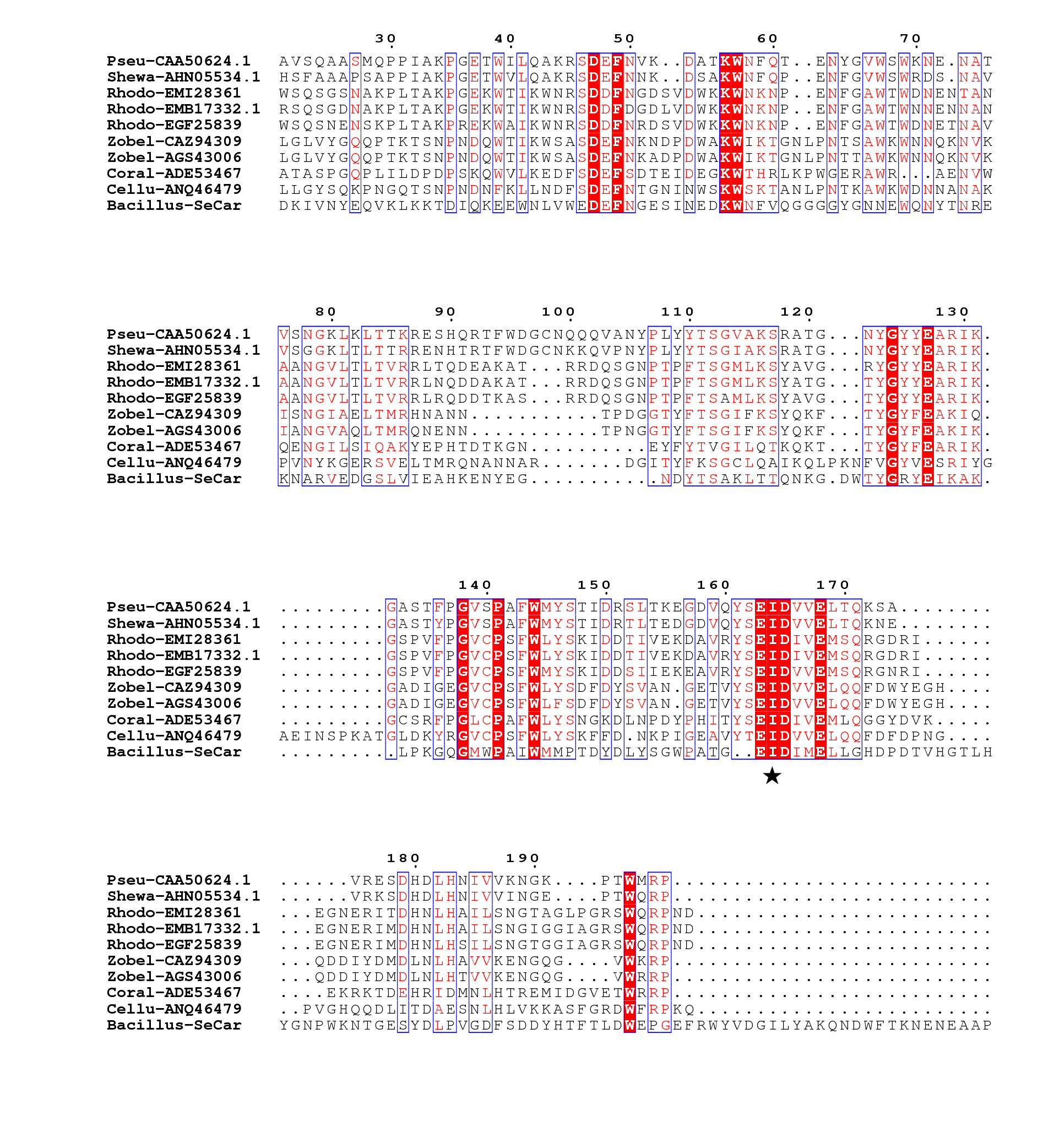

Supplement: Supplementary file 1 [file ijms-24-04672-s001.zip › Figure S1.tif]

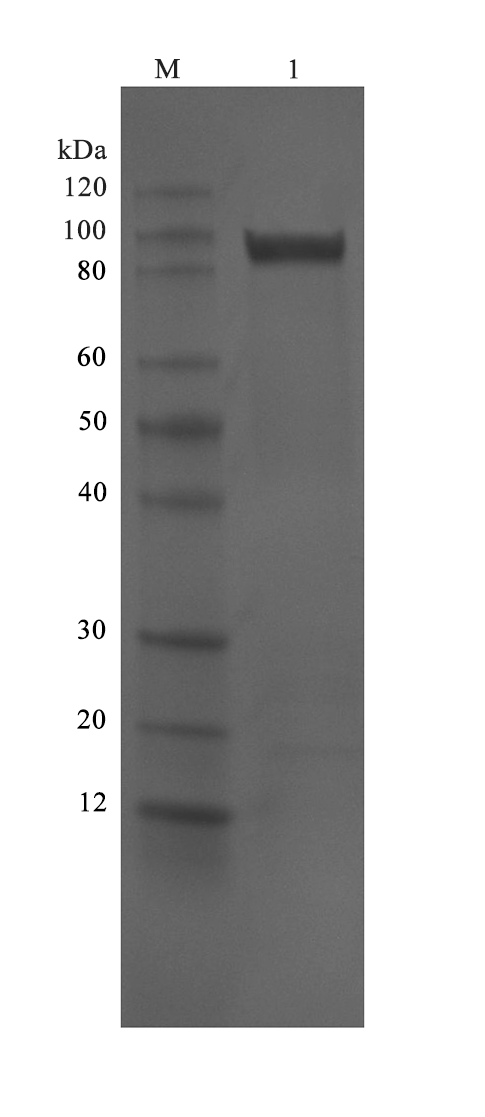

Supplement: Supplementary file 1 [file ijms-24-04672-s001.zip › Figure S2.tif]

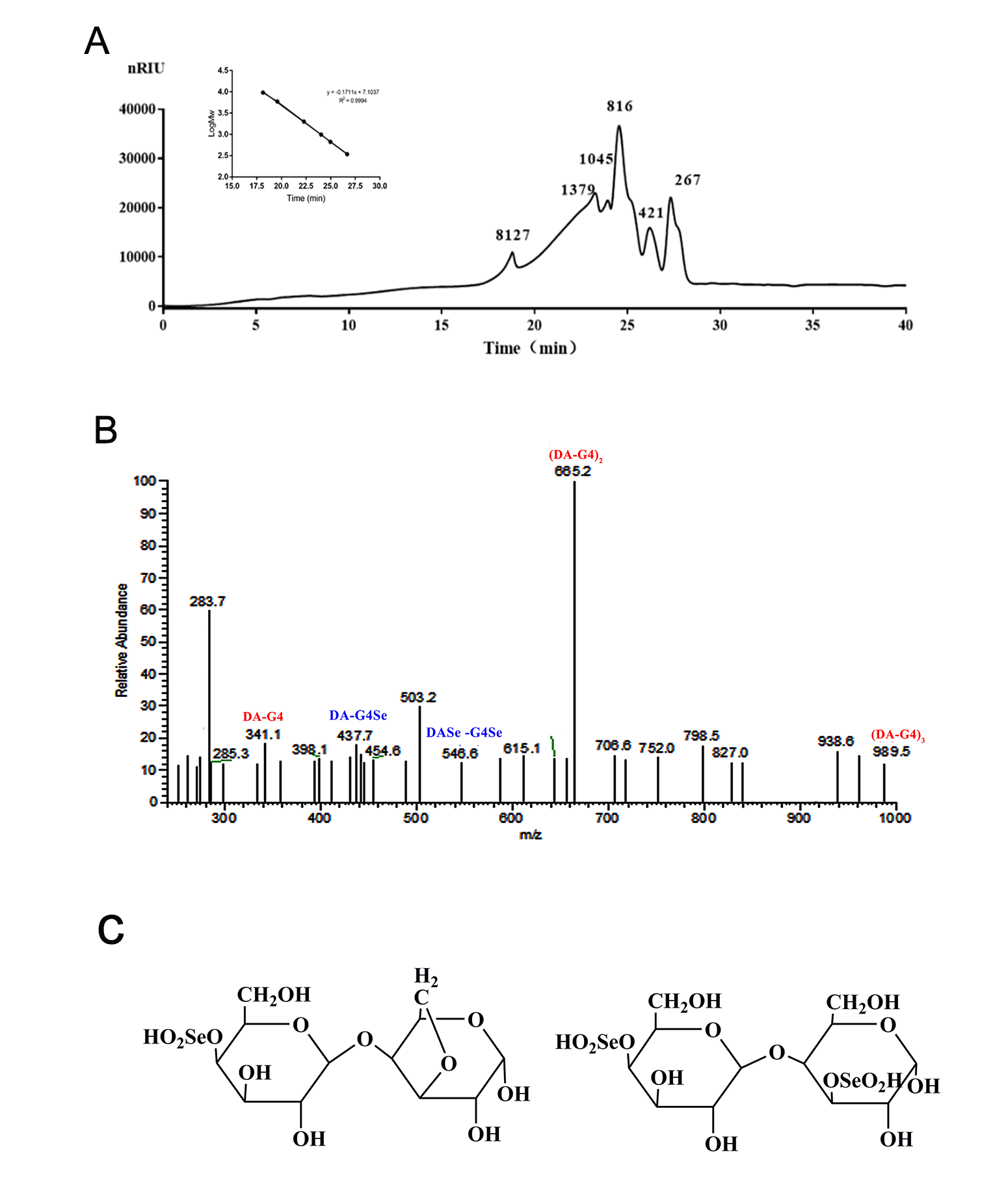

Supplement: Supplementary file 1 [file ijms-24-04672-s001.zip › Figure S3.tif]

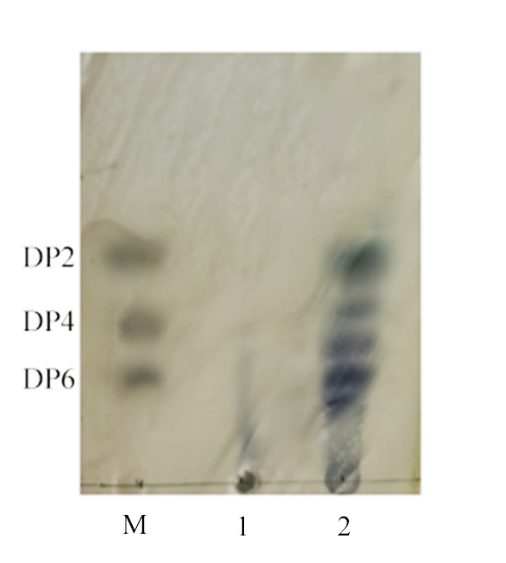

Supplement: Supplementary file 1 [file ijms-24-04672-s001.zip › Figure S4.tif]

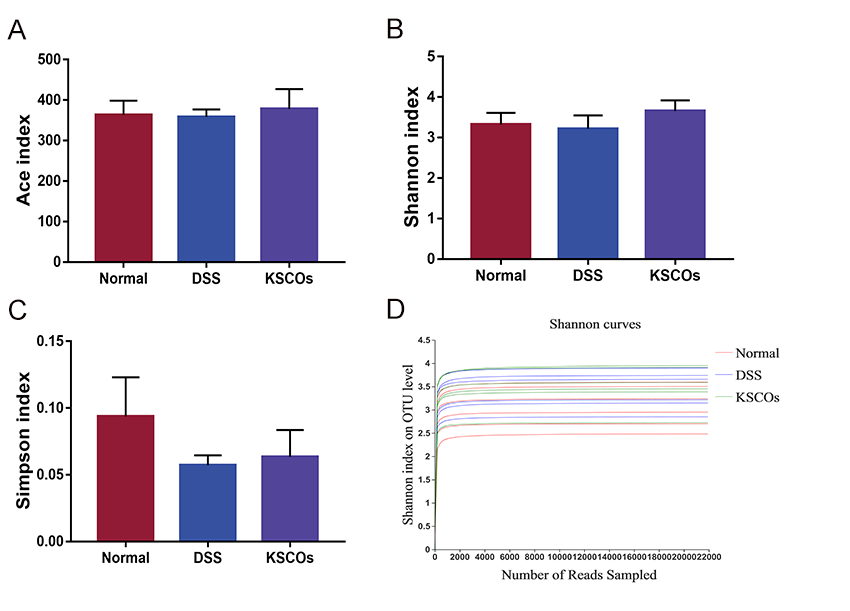

Supplement: Supplementary file 1 [file ijms-24-04672-s001.zip › Figure S5.tif]
